# Two-Dimensional DOA Estimation for Coherently Distributed Sources with Symmetric Properties in Crossed Arrays

**DOI:** 10.3390/s17061300

**Published:** 2017-06-06

**Authors:** Zhengliang Dai, Weijia Cui, Bin Ba, Daming Wang, Youming Sun

**Affiliations:** National Digital System Engineering and Technological Research R&D Center, Zhengzhou 450001, China; cuilink_work@sina.com (W.C.); xidianbabin@163.com (B.B.); wdm_wangdaming@foxmail.com (D.W.); sunyouming10@163.com (Y.S.)

**Keywords:** array signal processing, direction-of-arrival (DOA) estimation, coherently distributed (CD) sources, crossed array, symmetric property

## Abstract

In this paper, a novel algorithm is proposed for the two-dimensional (2D) central direction-of-arrival (DOA) estimation of coherently distributed (CD) sources. Specifically, we focus on a centro-symmetric crossed array consisting of two uniform linear arrays (ULAs). Unlike the conventional low-complexity methods using the one-order Taylor series approximation to obtain the approximate rotational invariance relation, we first prove the symmetric property of angular signal distributed weight vectors of the CD source for an arbitrary centrosymmetric array, and then use this property to establish two generalized rotational invariance relations inside the array manifolds in the two ULAs. Making use of such relations, the central elevation and azimuth DOAs are obtained by employing a polynomial-root-based search-free approach, respectively. Finally, simple parameter matching is accomplished by searching for the minimums of the cost function of the estimated 2D angular parameters. When compared with the existing low-complexity methods, the proposed algorithm can greatly improve estimation accuracy without significant increment in computation complexity. Moreover, it performs independently of the deterministic angular distributed function. Simulation results are presented to illustrate the performance of the proposed algorithm.

## 1. Introduction

In recent decades, the problem of direction-of-arrival (DOA) estimation, which plays an important role in radar, sonar and wireless communication systems, has attracted a lot of attention. The most commonly considered system model in the DOA finding techniques is the point source model, where the signals are assumed to arrive at the array via a single path [1,2,3,4]. When dealing with a point source, conventional subspace-based algorithms, such as the multiple signal classification (MUSIC) algorithm [5,6] and the estimation of signal parameters via rotational invariance techniques (ESPRIT) algorithm [7,8], have high DOA estimation resolution. However, in many practical applications, the signals will reach the array through many rays reflected or scattered from the vicinity, which causes angular spreading. In these cases, directly applying the MUSIC and ESPRIT algorithms may lead to biased estimations. Therefore, some researchers have considered a more realistic signal model called the spatially distributed source model [9,10]. Depending on the correlation among different rays, distributed sources are classified into two types: coherently and incoherently distributed (CD and ID) sources. In this paper, we only consider the DOA estimation of the CD sources.

Many DOA estimation techniques for CD sources have been published. Since conventional subspace-based methods cannot be directly applied to a distributed source, some researchers studied modifications to the MUSIC algorithm, which gave rise to the distributed signal parameter estimator [11], dispersed parametric estimator [12] and Vec-MUSIC estimator [13]. These three methods were established based on the assumption that the distribution shapes of multiply distributed sources are identical and known. In addition, the computational complexity is high because of the need for a two-dimensional (2D) search. The literature [14] used an unstructured model for the part of covariance matrix, where the 2D search problem was replaced by a successive one-dimensional (1D) search. With even lower computational complexity, a search-free algorithm called the spread root-MUSIC algorithm [15] was proposed to fit a two-ray model of the data. However, low-complexity was obtained in the special case where only one distributed source existed. In [16], the authors considered two identical and closely spaced sub-arrays. When the distance between the two sub-arrays was far shorter than the wavelength, an approximate rotational invariance relation between the two sub-arrays was obtained based on Taylor series approximation, and finally the central DOA of the CD source can be estimated by total least square estimation parameter via rotational invariance techniques (TLS-ESPRIT) using the generalized array manifold (GAM) model. All of the aforementioned works [11,12,13,14,15,16] were designed for 1D DOA and angular spread estimation of distributed sources. However, when the distributed source and the receiving sensor array are not in the same plane, it is reasonable to instead model the source as a 2D distributed source.

Since a 2D CD source is characterized by four parameters: the central azimuth direction, the azimuth angular extension, the central elevation direction and the elevation angular extension, the conventional optimum estimators will be computationally expensive owing to high-dimensional parameters [17]. Consequently, it is very necessary for 2D distributed sources to find some suboptimum algorithms to reduce the computational cost. To date, several low-complexity DOA estimation algorithms for 2D CD sources have been proposed. Specifically, the authors in [18] considered a pair of uniform circular arrays (UCAs). Preliminary estimations of central elevation DOAs were obtained using TLS-ESPRIT. Next, by using the estimated elevation DOAs, a sequential one-dimensional searching (SOS) method was proposed to estimate the central azimuth DOAs. In [19], using two parallel uniform linear arrays (ULAs), a low-complexity algorithm without searching was proposed for CD sources. Similarly, central elevation DOAs are obtained based on the approximate rotational invariance relation between the two ULAs. Instead of SOS, the quadric rotational invariance property (QRIP) of the GAM was used to estimate the central azimuth DOAs. Finally, a parameter matching approach was given to obtain the correct DOA estimation. In [20], the central elevation and azimuth DOAs were both estimated based on TLS-ESPRIT, which used two parallel ULAs, and the parameter matching method was also required. In order to avoid the parameter matching procedure, the literature [21] estimated the central elevation and central azimuth DOAs by applying the singular value decomposition method to the cross-correlation (CC) matrix of the received data in the double parallel ULAs. However, all the algorithms in [18,19,20,21] were all based on the special array geometry composed of two sub-arrays. The approximate rotational invariance relation between the two sub-arrays was obtained by using the one-order Taylor series approximation, which may introduce additional errors and affect the estimation accuracy.

In array processing, the crossed array is a commonly used 2D array geometry [22]. Compared to other 2D arrays such as the UCA and plane rectangular array, the crossed array can provide a larger aperture and hence offer better resolution for a given number of elements. Moreover, the crossed array consists of two intersecting ULAs working independently, thus the computational complexity is only double that of a single dimensional array. Several 2D estimation algorithms based on the crossed array have been proposed [23,24]. However, they are all based on the point source model. To the best of our knowledge, there have been few reports about the DOA estimation for CD sources in a crossed array.

In this paper, we consider a crossed array and divide it into two sub-ULAs. In particular, instead of using the Taylor series approximation, we prove the symmetric property of the angular signal distributed weight (ASDW) vector for an arbitrary centrosymmetric array, and use this property to establish the generalized rotational invariance relations inside the GAMs for the two sub-ULAs. Resorting to such relations, the central elevation and azimuth DOAs are estimated based on polynomial-root-based search-free method, respectively. Then simple parameter matching is accomplished by searching the minimums of the cost function of the estimated 2D angular parameters. The proposed algorithm does not require that the angular distribution functions of the multiple distributed sources are the same and known. In addition, it does not suffer additional errors induced by Taylor series approximation and high computational complexity brought about by spectrum-peak searching.

The rest of this paper is organized as follows: Section 2 presents the data model. In Section 3, we describe the proposed algorithm in detail. Some simulation results which illustrate the validity and performance of the proposed method are given in Section 4. Section 5 concludes the paper.

The following notations will be used throughout this paper. Superscript ${\left(\cdot \right)}^{*}$, ${\left(\cdot \right)}^{T}$, and ${\left(\cdot \right)}^{H}$ represent the conjugate, transpose and conjugate transpose operations, respectively. The symbol ⊗ denotes the Schur-Hadamard product; E[⋅] stands for the mathematical expectation and det(⋅) is the matrix determinant; diag[⋅] is a diagonal matrix and the values in the brackets are the diagonal elements.

## 2. Signal Model

**Figure 1:**
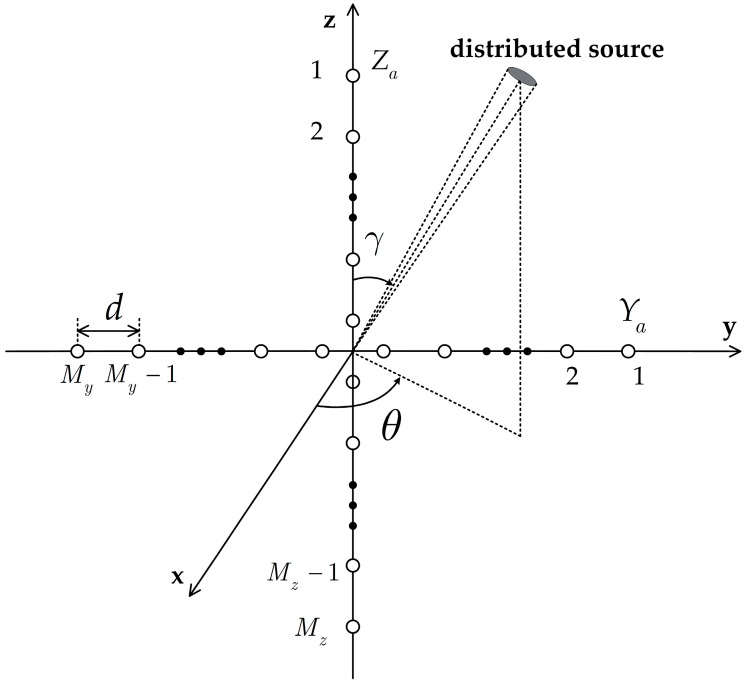
Geometry of the considered crossed array.

Let us consider the plane crossed array presented in Figure 1. The array is centered at the origin of the three-dimensional coordinate system with two ULAs directed along the *y*-axis and *z*-axis. The ULAs $Y_{a}$ and $Z_{a}$ are composed of $M_{y}$ and $M_{z}$ omni-directional antenna elements, respectively. The distance between adjacent sensors is d in the two ULAs. We assume that there are D narrowband CD sources impinging on the crossed array. The observation vectors of $Y_{a}$ and $Z_{a}$ at time t are given by [17,18,19,20,21].

(1) $$y(t)=\sum_{i=1}^{D}\iint a_{y}(\theta ,\gamma )s_{i}(\theta ,\gamma ,t;\mu_{i})d\theta d\gamma +n_{y}(t),$$

(2) $$z(t)=\sum_{i=1}^{D}\iint a_{z}(\theta ,\gamma )s_{i}(\theta ,\gamma ,t;\mu_{i})d\theta d\gamma +n_{z}(t),$$

where y(t) is the $M_{y}\times 1$ array output vector of the sub-array $Y_{a}$; z(t) is the $M_{z}\times 1$ array output vector of the sub-array $Z_{a}$; $s_{i}(\theta ,\gamma ,t;\mu_{i})$ is the complex random angular signal density function of the *i*-th source. The vector $\mu_{i}=(\theta_{i},\sigma_{\theta_{i}},\gamma_{i},\sigma_{\gamma_{i}})$ determines the central azimuth DOA $\theta_{i}$, the azimuth angular spread $\sigma_{\theta_{i}}$, the central elevation DOA $\gamma_{i}$ and the elevation angular spread $\sigma_{\gamma_{i}}$ of the *i*-th sensor; $n_{y}(t)$ and $n_{z}(t)$ are Gaussian white noise with zero-mean and variance, while $\sigma_{n}^{2}$; $a_{y}(\theta ,\gamma )$ and $a_{z}(\theta ,\gamma )$ are the two array manifold vectors at direction (θ,γ):

(3) $$\begin{array}{l} a_{y}(\theta ,\gamma )={\left[e^{j\eta 0.5(M_{y}-1)\sin\theta \sin\gamma },e^{j\eta 0.5(M_{y}-3)\sin\theta \sin\gamma },\ldots ,\;e^{-j\eta 0.5(M_{y}-1)\sin\theta \sin\gamma }\right]}^{T},\\ a_{z}(\theta ,\gamma )={\left[e^{j\eta 0.5(M_{z}-1)\cos\gamma },e^{j\eta 0.5(M_{z}-3)\cos\gamma },\ldots ,e^{-j\eta 0.5(M_{z}-1)\cos\gamma }\right]}^{T}, \end{array}$$

where η=2πd/λ, and λ is the wavelength of the coming signal.

For a 2D CD source, the angular signal density function $s_{i}(\theta ,\gamma ,t;\mu_{i})$ can be written as:

(4) $$s_{i}(\theta ,\gamma ,t;\mu_{i})=s_{i}(t)\rho_{i}(\theta ,\gamma ;\mu_{i}),$$

where $s_{i}(t)$ is a random variable and $\rho_{i}(\theta ,\gamma ;\mu_{i})$ is the deterministic angular distribution function.

Define the GAM vectors of distributed source for subarray $Y_{a}$ and $Z_{a}$ as follows:

(5) $$\begin{array}{l} b_{y}(\mu_{i})=\iint a_{y}(\theta ,\gamma )\rho_{i}(\theta ,\gamma ;\mu_{i})d\theta d\gamma ,\\ b_{z}(\mu_{i})=\iint a_{z}(\theta ,\gamma )\rho_{i}(\theta ,\gamma ;\mu_{i})d\theta d\gamma . \end{array}$$

For small angular extensions, we have the following closed forms [11,20]:

(6) $$\begin{array}{l} b_{y}(\mu_{i})=a_{y}(\theta_{i},\gamma_{i})⊗g_{y}(\mu_{i}),\\ b_{z}(\mu_{i})=a_{z}(\theta_{i},\gamma_{i})⊗g_{z}(\mu_{i}), \end{array}$$

where $g_{y}(\mu_{i})$ and $g_{z}(\mu_{i})$ are the ASDW vectors. The observation vectors in (1) and (2) can be written as:

(7) $$\begin{array}{l} y(t)=B_{y}(\mu )s(t)+n_{y}(t),\\ z(t)=B_{z}(\mu )s(t)+n_{z}(t), \end{array}$$

where $s(t)={\left[s_{1}(t),s_{2}(t),\ldots ,s_{D}(t)\right]}^{T}$ is a D×1 signal vector, and $B_{y}(\mu )$ and $B_{z}(\mu )$ are the GAM matrices, which are composed of *D* GAM vectors:

(8) $$\begin{array}{l} B_{y}(\mu )=[b_{y}(\mu_{1}),b_{y}(\mu_{2}),\ldots ,b_{y}(\mu_{D})],\\ B_{z}(\mu )=[b_{z}(\mu_{1}),b_{z}(\mu_{2}),\ldots ,b_{z}(\mu_{D})]. \end{array}$$

The total array output vector is expressed as:

(9) $$x(t)=\left[\begin{array}{l} y(t)\\ z(t) \end{array}\right].$$

## 3. The Proposed Algorithm

This section consists of four parts. Firstly, the symmetric property of an ASDW vector is identified in a centro-symmetric array. Then, by making use of the symmetric property of the ASDW vectors in the two sub-ULAs $Z_{a}$ and $Y_{a}$, we establish two generalized rotational invariance relations for the GAM vectors. On the premise of such relations, the central elevation and azimuth DOAs are obtained by using a polynomial-root-based search-free approach, respectively. Afterwards, and when multiple CD sources exist, a simple parameter matching approach is addressed. Finally, we provide the algorithm’s realization steps and an analysis of the computational complexity.

### 3.1. Symmetric Property of an ASDW Vector in a Centro-Symmetric Array

In this part, the symmetric property of an ASDW vector in a centro-symmetric array is derived in detail. Let us consider a centro-symmetric array consisting of *M* identical antenna elements centered at the coordinate origin, where the *m*-th sensor is placed at $\left(x_{m},y_{m},z_{m}\right)$ for m=1,2…,M. The array manifold vector in direction (θ,γ) is expressed as:

(10) $$a(\theta ,\gamma )=\left[\begin{array}{c} e^{j(2\pi /\lambda )(x_{1}\cos\theta \sin\gamma +y_{1}\sin\theta \sin\gamma +z_{1}\cos\gamma )}\\ e^{j(2\pi /\lambda )(x_{2}\cos\theta \sin\gamma +y_{2}\sin\theta \sin\gamma +z_{2}\cos\gamma )}\\ ......\\ e^{j(2\pi /\lambda )(x_{M}\cos\theta \sin\gamma +y_{M}\sin\theta \sin\gamma +z_{M}\cos\gamma )} \end{array}\right].$$

If we define $\theta =\theta_{i}+\tilde{\theta }$ and $\gamma =\gamma_{i}+\tilde{\gamma }$, in which $\theta_{i}$, $\gamma_{i}$ are the central azimuth DOA and the central elevation DOA of the *i*-th source, and $\tilde{\theta }$, $\tilde{\gamma }$ are the corresponding random angular deviations , the GAM vector can be presented as:

(11) $$\begin{array}{cc} b(\mu_{i}) & =\iint a(\theta ,\gamma )\rho (\theta ,\gamma ;\mu_{i})d\theta d\gamma \\ & =\iint a(\theta_{i}+\tilde{\theta },\gamma_{i}+\tilde{\gamma })\rho (\tilde{\theta },\tilde{\gamma };\mu_{i})d\tilde{\theta }d\tilde{\gamma }, \end{array}$$

For small angular extensions, the *m*-th element of $b(\mu_{i})$ can be written as [11]:

(12) $${\left[b(\mu_{i})\right]}_{m}\approx {\left[a(\theta_{i},\gamma_{i})\right]}_{m}\cdot \iint e^{j\varsigma_{m}}\rho (\tilde{\theta },\tilde{\gamma };\mu_{i})d\tilde{\theta }d\tilde{\gamma }.$$

Thus, the *m*-th element of ASDW vector is given by:

(13) $${\left[g(u)\right]}_{m}=\iint e^{j\varsigma_{m}}\rho (\tilde{\theta },\tilde{\gamma };\mu_{i})d\tilde{\theta }d\tilde{\gamma },$$

where:

(14) $$\begin{array}{l} \varsigma_{m}=(2\pi /\lambda )[x_{m}(-\tilde{\theta }\sin\theta_{i}\sin\gamma_{i}+\tilde{\gamma }\cos\theta_{i}\cos\gamma_{i})+\;\\ y_{m}(\tilde{\theta }\cos\theta_{i}\sin\gamma_{i}+\tilde{\gamma }\sin\theta_{i}\cos\gamma_{i})+z_{m}(-\tilde{\gamma }\sin\gamma_{i})]. \end{array}$$

We have $\left(x_{m},y_{m},z_{m})=-(x_{m+M/2},y_{m+M/2},z_{m+M/2}\right)$ in the centrosymmetric array, thus $\varsigma_{m}=-\varsigma_{m+M/2}$. Respecting the fact that $\rho (\tilde{\theta },\tilde{\gamma };\mu_{i})$ is an even function (see Appendix A), we can obtain the symmetric property of the ASDW vector such as:

(15) $${\left[g(u)\right]}_{m}={\left[g(u)\right]}_{m+M/2}.$$

It is obvious that $Y_{a}$ and $Z_{a}$ are centrosymmetric arrays, thus we have:

(16) $$\begin{array}{l} {\left[g_{y}(u)\right]}_{m}={\left[g_{y}(u)\right]}_{m+M/2},\\ {\left[g_{z}(u)\right]}_{m}={\left[g_{z}(u)\right]}_{m+M/2}. \end{array}$$

### 3.2. Derivation

#### 3.2.1. Central Elevation DOA Estimation

For sub-array $Z_{a}$, and owing to the symmetric property of the ASDW vector in (16), we can establish the following generalized rotational invariance relation of the GAM vector:

(17) $$\Pi_{M_{Z}}b_{z}(\mu_{i})=\Psi (\gamma_{i})b_{z}(\mu_{i}),$$

where $\Pi_{M_{Z}}$ is the $M_{z}\times M_{z}$ exchange matrix with ones on its anti-diagonal and zeros elsewhere. $\Psi (\gamma_{i})$ is an $M_{z}\times M_{z}$ diagonal matrix which is given by:

(18) $$\Psi (\gamma_{i})=diag[e^{-j\eta (M_{z}-1)\cos\gamma_{i}},e^{-j\eta (M_{z}-3)\cos\gamma_{i}},\ldots ,e^{j\eta (M_{z}-1)\cos\gamma_{i}}].$$

If we define the complex variable $k=e^{j\eta \cos\gamma_{i}}$, $\Psi (\gamma_{i})$ can be written as:

(19) $$\Psi (k)=diag[k^{-(M_{z}-1)},k^{-(M_{z}-3)},\ldots ,k^{\left(M_{z}-1\right)}].$$

According to the observation vector z(t) in (7), the covariance matrix of z(t) is expressed as:

(20) $$R_{z}=E\{z(t)z^{H}(t)\}=B_{y}(\mu )R_{s}B_{y}^{H}(\mu )+\sigma_{n}^{2}I_{M_{z}},$$

where $R_{s}=E\{s(t)s^{H}(t)\}$ is the signal covariance matrix of the CD sources. The eigenvalue decomposition of $R_{z}$ is given by:

(21) $$R_{z}=U_{z}\Sigma_{z}U_{z}^{H}+U_{n}\Sigma_{n}U_{n}^{H},$$

where $U_{z}$ is the signal subspace matrix, whose columns are the eigenvectors corresponding to the D largest eigenvalues of $R_{z}$. When $R_{s}$ is of full rank, the subspace spanned by the columns of $U_{z}$ is equal to the subspace spanned by the columns of $B_{z}(\mu )$. At this point, there exists a unique non-singular D×D matrix $T_{1}$ such that $U_{z}=B_{z}(\mu )T_{1}$. According to the generalized rotational invariance relation in (17), we can formulate a matrix $F_{z}(k)$:

(22) $$\begin{array}{cc} F_{z}(k) & =\Pi_{M_{Z}}U_{z}-\Psi (k)U_{z}\\ & =\Pi_{M_{Z}}B_{z}(\mu )T_{1}-\Psi (k)B_{z}(\mu )T_{1}\\ & =[(\Psi (k_{1})-\Psi (k))b_{z}(\mu_{1}),(\Psi (k_{2})-\Psi (k))b_{z}(\mu_{2}),\\ & \;\ldots ,(\Psi (k_{D})-\Psi (k))b_{z}(\mu_{D})]T_{1}. \end{array}$$

Therefore, when $\Psi (k_{i})=\Psi (k)$ for i=1,2,…,D, the *i*-th column of $F_{z}(k)$ is a zero vector. Hence, $F_{z}(k)$ is rank deficient and the determinant of $F_{z}^{H}(k)F_{z}(k)$ is zero. The central elevation DOA estimations ${\hat{\gamma }}_{i}(i=1,2\ldots ,D)$ can be obtained by finding the highest D peaks of the following spectrum function:

(23) $$H_{z}(k)=1/\det(F_{z}^{H}(k)F_{z}(k)).$$

However, estimator (23) involves computationally intensive spectral-peak searching. In order to reduce the complexity, we derive a polynomial-root-based search-free approach. The denominator of (23) can be written as the following polynomial:

(24) $$h_{z}(k)=\det(F_{z}^{H}(k)F_{z}(k)).$$

It is obvious that the central elevation DOAs can be obtained by rooting this polynomial. Note that the roots of (24) appear in conjugate reciprocal pairs, as in the conventional root-MUSIC [25]. To find the D central elevation DOAs, we select the D roots $k_{i}(i=1,2\ldots ,D)$ inside the unit circle that maximize (23). Finally, the central elevation DOA estimates are obtained by:

(25) $${\hat{\gamma }}_{i}=ar\cos\left(\frac{\lambda }{2\pi d}\arg(k_{i})\right).$$

#### 3.2.2. Central Azimuth DOA Estimation

The method is similar to that of the central elevation DOA estimate, thus we simplify the process of deduction. For the centrosymmetric sub-array $Y_{a}$, we also have the generalized shift invariance relation:

(26) $$\Pi_{M_{y}}b_{y}(\mu_{i})=\Omega (\theta_{i},\varphi_{i})b_{y}(\mu_{i}),$$

where $\Omega (\theta_{i},\varphi_{i})$ is a $M_{y}\times M_{y}$ diagonal matrix which is given by:

(27) $$\begin{array}{cc} \Omega (\theta_{i},\varphi_{i}) & =diag[e^{-j\eta (M_{y}-1)\sin\theta_{i}\sin\gamma_{i}},e^{-j\eta (M_{y}-3)\sin\theta_{i}\sin\gamma_{i}},\\ & \;\ldots ,e^{j\eta (M_{y}-1)\sin\theta_{i}\sin\gamma_{i}}]. \end{array}$$

If we define the complex variable $l=e^{j\eta \sin\theta_{i}\sin\gamma_{i}}$, $\Omega (\theta_{i},\varphi_{i})$ can be written as: $\Omega (l)=diag[l^{-(M_{y}-1)},l^{-(M_{y}-3)},\ldots ,l^{\left(M_{y}-1\right)}]$.

Let $U_{y}$ be the signal subspace matrix, whose columns are the eigenvectors corresponding to the D largest eigenvalues of $R_{y}=E\{y(t)y^{H}(t)\}$. Similarly, there exists a unique non-singular D×D matrix $T_{2}$ such that $U_{y}=B_{y}(\mu )T_{2}$. Thus, we introduce a matrix $F_{y}(l)$ which is expressed as:

(28) $$\begin{array}{cc} F_{y}(l) & =\Pi_{M_{y}}U_{y}-\Omega (l)U_{y}\\ & =\Pi_{M_{y}}B_{y}(\mu )T_{3}-\Omega (l)B_{y}(\mu )T_{2}\\ & =[(\Omega (l_{1})-\Omega (l))b_{y}(\mu_{1}),(\Omega (l_{2})-\Omega (l))b_{y}(\mu_{2}),\\ & \;\ldots ,(\Omega (l_{D})-\Omega (l))b_{y}(\mu_{D})]T_{2}. \end{array}$$

The central azimuth DOA estimations ${\hat{\theta }}_{i}(i=1,2\ldots ,D)$ can be obtained by rooting the following polynomial:

(29) $$h_{y}(l)=\det\{F_{y}^{H}(l)F_{y}(l)\}.$$

Similarly, we use the roots inside the unit circle, and select the D roots $l_{i}(i=1,2\ldots ,D)$ that maximize the spectral function such as:

(30) $$H_{y}(l)=1/\det(F_{y}^{H}(l)F_{y}(l)).$$

The values of $\sin{\hat{\theta }}_{i}\cdot \sin{\hat{\gamma }}_{i}$ for i=1,2,…,D are obtained as:

(31) $$\sin{\hat{\theta }}_{i}\cdot \sin{\hat{\gamma }}_{i}=\frac{\lambda }{2\pi d}\arg(l_{i}).$$

#### 3.2.3. The Parameter Matching Method

For only one CD source, the central elevation and azimuth DOAs can be estimated directly using (25) and (31). However, when multiple CD sources exist, the estimated elevation and azimuth DOAs are required to be matched. To perform the pair-matching procedure, we need to consider the GAM vector of the whole cross array such as:

(32) $$b_{x}(\mu_{i})=\left[\begin{array}{l} b_{y}(\mu_{i})\\ b_{z}(\mu_{i}) \end{array}\right].$$

Let J be an $\left(M_{y}+M_{z})\times (M_{y}+M_{z}\right)$ selection matrix which is defined as:

(33) $$J=\left[\begin{array}{cc} \Pi_{M_{y}} & 0_{M_{y}\times M_{Z}}\\ 0_{M_{z}\times M_{y}} & \Pi_{M_{Z}} \end{array}\right].$$

Based on the symmetric property of the ASDW vector in (16), we have the following generalized rotational invariance relation:

(34) $$Jb_{x}(\mu_{i})=\Phi (\theta_{i},\gamma_{i})b_{x}(\mu_{i}),$$

where $\Phi (\theta_{i},\gamma_{i})$ is an $\left(M_{y}+M_{z})\times (M_{y}+M_{z}\right)$ diagonal matrix given by:

(35) $$\begin{array}{l} \Phi (\theta_{i},\gamma_{i})=diag[e^{-j\eta (M_{y}-1)\sin\theta_{i}\sin\gamma_{i}},e^{-j\eta (M_{y}-3)\sin\theta_{i}\sin\gamma_{i}},\ldots ,\\ e^{j\eta (M_{y}-1)\sin\theta_{i}\sin\gamma_{i}},e^{-j\eta (M_{z}-1)\cos\gamma_{i}},e^{-j\eta (M_{z}-3)\cos\gamma_{i}},\ldots ,e^{j\eta (M_{z}-1)\cos\gamma_{i}}]. \end{array}$$

Let $U_{x}$ be the signal subspace matrix of $R_{x}=E\{x(t)x^{H}(t)\}$. Similarly, there exists a unique non-singular D×D matrix $T_{3}$ such that $U_{x}=B_{x}(\mu )T_{3}$. We can also introduce a matrix $F_{x}(\theta ,\gamma )$ which is written as:

(36) $$\begin{array}{cc} F_{x}(\theta ,\gamma ) & =JU_{x}-\Phi (\theta ,\gamma )U_{x}\\ & =JB_{x}(\mu )T_{2}-\Phi (\theta ,\gamma )B_{x}(\mu )T_{3}\\ & =[(\Phi (\theta_{1},\gamma_{1})-\Phi (\theta ,\gamma ))b_{x}(\mu_{1}),(\Phi (\theta_{2},\gamma_{2})-\Phi (\theta ,\gamma ))b_{x}(\mu_{2}),\\ & \;\ldots ,(\Phi (\theta_{D},\gamma_{D})-\Phi (\theta ,\gamma ))b_{x}(\mu_{D})]T_{3}. \end{array}$$

Similarly, when $\Phi (\theta_{i},\gamma_{i})=\Phi (\theta ,\gamma )$ for i=1,2,…,D, the *i*-th column of $F_{x}(\theta ,\gamma )$ is a zero vector. Therefore, the central elevation and azimuth DOA estimations can be matched by minimizing of the following cost function:

(37) $$f(\theta ,\gamma )=\det(F_{x}^{H}(\theta ,\gamma )F_{x}(\theta ,\gamma )).$$

If we pick ${\hat{\gamma }}_{i}$ from the elevation DOA estimations $\left\{{\hat{\gamma }}_{1},{\hat{\gamma }}_{2},\ldots {\hat{\gamma }}_{D}\right\}$, there will be D pairs of 2D central DOAs for ${\hat{\gamma }}_{i}$, which is given by $\left\{({\hat{\theta }}_{i1,}{\hat{\gamma }}_{i}),({\hat{\theta }}_{i2,}{\hat{\gamma }}_{i}),\ldots ({\hat{\theta }}_{iD,}{\hat{\gamma }}_{i})\right\}$. We then substitute the DOA estimations into (37) and calculate the function value f(θ,γ). If $f({\hat{\theta }}_{ij},{\hat{\gamma }}_{i})$ for j=1,2,…D is the largest, then $\left({\hat{\theta }}_{ij},{\hat{\gamma }}_{i}\right)$ is the correct match.

#### 3.2.4. Algorithm Implementation and Complexity Analysis

Now, we can summarize the proposed algorithm as follows:

Step 1: Calculate the covariance matrix $R_{z}=E\{z(t)z^{H}(t)\}$. Through the eigen-decomposition of $R_{z}$, obtain the signal subspace matrix $U_{z}$.
Step 2: Construct the matrix $F_{z}(k)$ in (22), and root the polynomial in (24) to obtain the central elevation DOA estimations ${\hat{\gamma }}_{i}$ for i=1,2,…D. It is noted that the roots are inside a unit circle and maximize (23).
Step 3: Calculate the covariance matrix $R_{y}=E\{y(t)y^{H}(t)\}$. Through the eigen-decomposition of $R_{y}$, obtain the signal subspace matrix $U_{y}$.
Step 4: Construct the matrix $F_{y}(k)$ in (28), and root the polynomial in (29) to obtain $\sin{\hat{\theta }}_{i}\cdot \sin{\hat{\gamma }}_{i}$ for i=1,2,…D. It is noted that the roots are inside a unit circle and maximize (30).
Step 5: Compute all the possible 2D DOAs $\left({\hat{\theta }}_{ij},{\hat{\gamma }}_{i}\right)$ for the elevation DOA estimations ${\hat{\gamma }}_{i}$. Calculate the function values $f({\hat{\theta }}_{ij},{\hat{\gamma }}_{i})$ for j=1,2,…D in (37). The largest one is the correct match.
Step 6: Repeat the process in Step 5 to match all the parameters.

Next, when the number of sensor elements *M and* the number of snapshots *L* change, we analyze the computational complexity of the proposed algorithm in comparison with the SOS algorithm in [18], the CC algorithm in [21] and Zheng’s algorithm in [20]. The main computational cost of the proposed algorithm is mostly made of four operations: the estimation of the covariance matrix, the eigen-decomposition of the covariance matrix, the polynomials rooting, and the pair-matching procedure. Specifically, the cost involved by the estimation of covariance matrices $R_{x}$, $R_{y}$ and $R_{z}$ is found to be in $O(6M^{2}L)$. The eigen-decomposition of the covariance matrices $R_{x}$, $R_{y}$ and $R_{z}$ needs $O(10M^{3})$ multiplications. The computational complexity of rooting polynomials $h_{z}(k)$ and $h_{y}(k)$ is found to be in $O(2M^{3})$, and the pair-matching procedure adds $O(D^{5}+D^{4}M)$ multiplications to the proposed algorithm. In above, the computational complexity of the proposed algorithm is $O(12M^{3}+6M^{2}L+D^{5}+D^{4}M)$. Moreover, the main computational complexity of the SOS algorithm, the CC algorithm and Zheng’s algorithm is given in Table 1 (*N denotes* the number of searching points).

When the number of searching points *N* is large, it is clear to see that the propose algorithm provides lower computational cost compared to the SOS algorithm. Although the computational complexity of the proposed algorithm is higher than Zheng’s algorithm and the CC algorithm, it is not significant increment since the proposed algorithm does not require any spectrum searching. In addition, and unlike the SOS algorithm, Zheng’s algorithm and the CC algorithm, the proposed algorithm does not use the Taylor series approximation to establish the rotational invariance relation, as this approximation may introduce additional errors.

## 4. Simulation Results and Performance Analysis

In the following experiments, noise is a complex Gaussian process with zero mean. The number of snapshots is 200. We use the root mean squared error (RMSE) to evaluate the estimation performance, where the RMSE of the central azimuth and elevation DOAs (RMSE(θ) and RMSE(γ)) are defined as:

(38) $$\mathrm{RMSE}(\theta )=\sqrt{E[\frac{1}{D}\sum_{i=1}^{D}{\left({\hat{\theta }}_{i}-\theta_{i}\right)}^{2}]},$$

(39) $$\mathrm{RMSE}(\gamma )=\sqrt{E[\frac{1}{D}\sum_{i=1}^{D}{\left({\hat{\gamma }}_{i}-\gamma_{i}\right)}^{2}]},$$

where ${\hat{\theta }}_{i}$ and $\theta_{i}$ are the estimated and true central azimuth DOA of the *i*-th source, respectively. Additionally, ${\hat{\gamma }}_{i}$ and $\gamma_{i}$ are the estimated and true central elevation DOA of the *i*-th source, respectively.

In the following simulations, the signal power of sources is assumed to be the same. In addition, signal-to-noise ratio (SNR) is defined as:

(40) $$\mathrm{SNR}=10\log\frac{\sigma_{s}^{2}}{\sigma_{n}^{2}},$$

where $\sigma_{s}^{2}$ is the signal power of sources, while $\sigma_{n}^{2}$ is the variance of noise.

### 4.1. Effect of Different Deterministic Angular Distributed Functions

**Figure 2:**
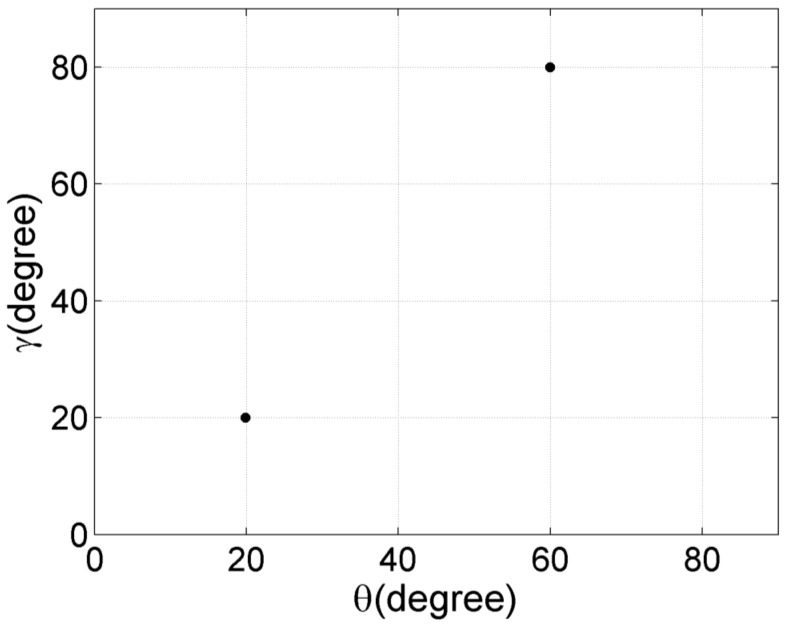
The 2D central DOA estimation results of the proposed algorithm (30 trials).

In this part, we examine if the proposed algorithm works properly for different angular distributed functions. The sub-arrays $Y_{a}$ and $Z_{a}$ are both composed of $M_{y}=M_{z}=16$ sensors. The distance between adjacent sensors is 0.5λ. The parameters of two CD sources are $\mu_{1}=(20{}^{\circ},3{}^{\circ},20{}^{\circ},5{}^{\circ})$ and $\mu_{2}=(60{}^{\circ},4{}^{\circ},80{}^{\circ},4{}^{\circ})$. Their deterministic angular distributed functions are Gaussian and uniform shaped, respectively. The SNR is 15 dB. For 30 independent trials, the central DOA estimations of CD sources are plotted in Figure 2. It can be seen that the proposed algorithm can give the correct DOA estimations for cases where different CD sources have different deterministic angular distributed functions, or unknown deterministic angular distributed functions.

### 4.2. Performance Comparison

**Figure 3:**
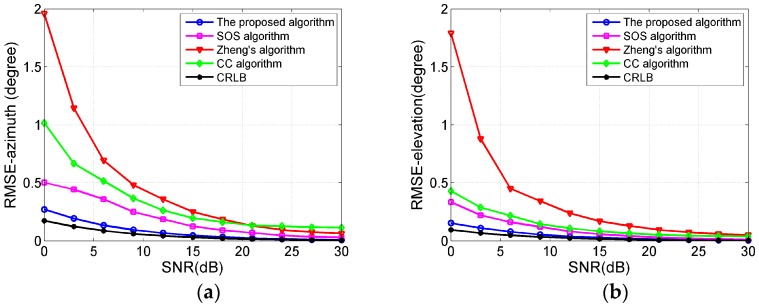
(**a**) RMSE curves of the central azimuth DOA estimations versus SNR; (**b**) RMSE curves of the central elevation DOA estimations versus SNR.

In this part, we compare the estimation accuracy of the proposed algorithm with the SOS algorithm in [18], the CC algorithm in [21] and Zheng’s algorithm in [20] with respect to SNR from 0 dB to 30 dB. The Cramér-Rao lower bound (CRLB) is also used as a benchmark [26]. The sub-arrays $Y_{a}$ and $Z_{a}$ of the crossed array in proposed algorithm are both composed of $M_{y}=M_{z}=16$ sensors. The arrays in the SOS algorithm and the CC algorithm are both composed of 32 sensors. Since the number of antenna elements in Zheng’s algorithm must be odd, we set it to 33. In these algorithms, the distance between adjacent sensors in a sub-array is 0.5λ, the vertical distance between the two sub-arrays is 0.5λ, and the radius of the UCA is λ/(4sin(π/16)). The parameters of two Gaussian-shaped CD sources are $\mu_{1}=(20{}^{\circ},2{}^{\circ},60{}^{\circ},3{}^{\circ})$ and $\mu_{2}=(15{}^{\circ},3{}^{\circ},70{}^{\circ},2{}^{\circ})$, respectively. Based on 500 Monte Carlo experiments, the RMSE curves of the central DOA estimations versus SNR are shown in Figure 3. It is clearly indicated that the estimation accuracy of the proposed algorithm is higher than the SOS algorithm, the CC algorithm and Zheng’s algorithm, which arises from the fact that the proposed algorithm does not suffer additional errors brought about by Taylor series approximation.

### 4.3. Effect of Snapshots

**Figure 4:**
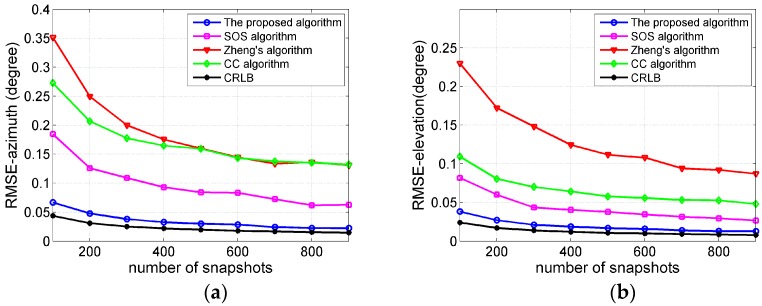
(**a**) RMSE curves of the central azimuth DOA estimations versus the number of snapshots; (**b**) RMSE curves of the central elevation DOA estimations versus the number of snapshots.

In this part, we illustrate the influence of the number of snapshots on the performance of the proposed algorithm. The number of snapshots varies from 100 to 900. The SNR is fixed to 15 dB and the other parameters are the same as in Section 4.2. Based on 500 Monte Carlo experiments, the RMSE curves for different algorithms are shown in Figure 4, from which we can draw similar conclusion as in Section 4.2.

### 4.4. Effect of the Central Elevation and Azimuth DOAs

**Figure 5:**
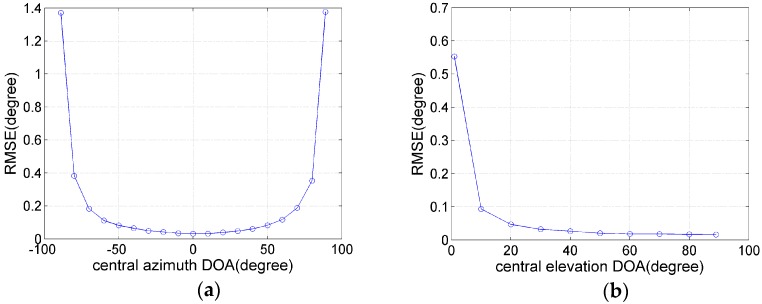
(**a**) RMSE curves versus the central azimuth DOA; (**b**) RMSE curves versus the central elevation DOA.

In the last example, we examine the performance of the proposed method versus the central elevation and azimuth DOAs for a Gaussian-shaped CD source with $\sigma_{\theta_{i}}=\sigma_{\gamma_{i}}=1{}^{\circ}$. First, let us consider in Figure 5a the influence of the central azimuth DOA on performance. Assume that $\gamma_{i}=30{}^{\circ}$, SNR = 10 dB and the number of snapshots L=200. As can be seen from the Figure 5a, the RMSE of ${\hat{\theta }}_{i}$ estimated by the proposed method increases dramatically when the central azimuth DOA approaches the lower bound ($\theta_{i}=-90{}^{\circ}$) or the upper bound ($\theta_{i}=90{}^{\circ}$), but our method can still estimate effectively the central azimuth DOA. Next, the influence of the central elevation DOA on performance is examined in Figure 5b. At this time, we assume that $\theta_{i}=30{}^{\circ}$, SNR = 10 dB and L=200. Similarly, the RMSE of ${\hat{\gamma }}_{i}$ estimated by the proposed method also increases dramatically when the central elevation DOA approaches the lower bound ($\gamma_{i}=0{}^{\circ}$), and our method has still satisfying estimation performance for approaching the lower bound.

## 5. Conclusions

In this paper, we have presented a new approach for estimating the 2D central DOA of CD sources using a centrosymmetric crossed array. Instead of using the Taylor series approximation, we derive the symmetric property of an ASDW vector in a centrosymmetric array, based on which the generalized shift invariance relations inside the GAMs are established in the two sub-ULAs. Resorting to such relations, the central elevation and azimuth DOAs are estimated based on the polynomial-root-based search-free method, respectively. After that, the pair-matching method is presented. The proposed algorithm performs independently of the deterministic angular distributed function. Compared to the existing low-complexity algorithms, the proposed algorithm does not suffer additional errors brought by the Taylor series approximation, which allows it to achieve a higher estimation accuracy. Moreover, the proposed algorithm does not suffer high computational complexity brought by spectrum-peak searching.

## Figures and Tables

**Table 1:** Comparison of different algorithms in computational complexity.

Algorithm	Main Computational Complexity
Proposed algorithm	$O(12M^{3}+6M^{2}L+D^{5}+D^{4}M)$
SOS algorithm	$O(8M^{3}+4M^{2}L+N(D^{3}+3D^{2}M))$
CC algorithm	$O(M^{3}+M^{2}L+D^{3})$
Zheng’s algorithm	$O({\left(2M+1\right)}^{3}+{\left(2M+1\right)}^{2}L+D^{3})$

## Appendix A

The most commonly used the deterministic angular distributed functions for a 2D CD source are presented as follows:

Gaussian shaped:

(A1) $$\rho (\tilde{\theta },\tilde{\gamma };\mu_{i})=\frac{1}{2\pi \sigma_{\theta }\sigma_{\gamma }}e^{-\frac{1}{2}({\tilde{\theta }}^{2}/\sigma_{\theta }^{2}+{\tilde{\gamma }}^{2}/\sigma_{\gamma }^{2})},$$

Uniform shaped:

(A2) $$\rho (\tilde{\theta },\tilde{\gamma };\mu_{i})=\left\{ \begin{array}{ll} \frac{1}{12\sigma_{\theta }\sigma_{\gamma }} & \left|\tilde{\theta }\right|\leq \sqrt{3}\sigma_{\theta }\;and\;\left|\tilde{\gamma }\right|\leq \sqrt{3}\sigma_{\gamma }\\ 0 & \mathrm{otherwise} \end{array}\right.,$$

Laplacian shaped:

(A3) $$\rho (\tilde{\theta },\tilde{\gamma };\mu_{i})=\frac{1}{2\sigma_{\theta }\sigma_{\gamma }}e^{-(\sqrt{2}\left|\tilde{\theta }\right|/\sigma_{\theta }+\sqrt{2}\left|\tilde{\gamma }\right|/\sigma_{\gamma })},$$

where the small deviation $\tilde{\theta }$ and $\tilde{\gamma }$ are defined as $\tilde{\theta }=\theta -\theta_{i}$ and $\tilde{\gamma }=\gamma -\gamma_{i}$, respectively.
